# Incidence and risk factors of post COVID-19 syndrome: a Tunisian cohort study

**DOI:** 10.1186/s12879-023-08949-8

**Published:** 2024-05-01

**Authors:** Imen Zemni, Amel Gara, Cyrine Bennasrallah, Salma Ezzar, Meriem Kacem, Roua Chokri, Amani Maatouk, Hela Abroug, Wafa Dhouib, Manel Ben Fredj, Ines Bouanene, Asma Sriha Belguith

**Affiliations:** 1 Department of Epidemiology and Preventive Medicine, Fattouma Bourguiba University Hospital, Monastir, Tunisia; 2 Department of Epidemiology, Faculty of Medicine of Monastir, University of Monastir, Monastir, Tunisia; 3 Technology and Medical Imaging Research Laboratory - LTIM - LR12ES06, University of Monastir, Monastir, Tunisia; 4 Department of Family Medicine, Faculty of Medicine of Monastir, Monastir, Tunisia

**Keywords:** Post-acute COVID-19 syndrome, Risk factors, Incidence, Prospective studies, Tunisia

## Abstract

**Background:**

It has become increasingly clear that SARS-CoV-2 infection can lead to persistent physical and mental health problems lasting weeks or months, requiring prolonged periods of clinical care and increasing the burden on the healthcare system. This phenomenon, known as post COVID-19 syndrome (PCS), is a relatively new condition, its incidence is still unclear and differs between studies.

**Objectives:**

In this cohort study, we aimed to estimate the incidence of PCS and to identify its risk factors in the Tunisian population.

**Methods:**

This is a prospective cohort study that enrolled patients diagnosed with COVID-19 from the triage unit of the University Hospital of Monastir, Tunisia. between April 2021 and June 2022. Patients were contacted by phone for a follow-up evaluation of PCS 12- weeks after the diagnosis date.

**Results:**

A total of 1451 individuals diagnosed with COVID-19 during the study period, responded to the follow-up evaluation after 3 months. The incidence of PCS was found to be 44.03% (95% CI [41.47; 46.58]), with fatigue being the most common symptom (21.5%), followed by cognitive impairment (10.3%), including memory loss and difficulty concentrating. Multivariate analysis revealed that the main associated factors to PCS were female gender (RR = 1.54; CI95% [1.30 - 1.82]), pre-existing comorbidities (RR = 1.30; CI95% [1.10 - 1.52]), duration of acute COVID-19 illness (days) (RR = 1.02; CI95% [1.01 - 1.03]), hospitalization (RR = 1.27; CI95% [1.05 - 1.53]), number of COVID-19 episodes (RR = 1.46; CI 95% [1.28 - 1.67]) and patients having receive two or more doses of vaccine prior to COVID-19 infection (RR = 0.82; CI95% [0.70 - 0.96]).

**Conclusion:**

Our study allowed to estimate the incidence and identify risk factors of PCS. Recognizing these factors could help to better understand the underlying mechanisms and guide interventions for prevention and management of this condition.

## Introduction

For over 3 years now, SARS-CoV-2 infection (COVID-19) has affected almost every region of the world, with more than 700 million confirmed cases and more than 6 million lives lost, causing significant economic repercussions [1]. The disease presents a wide range of symptoms, spanning from asymptomatic or mild cases to severe respiratory problem, multi-organ failure, and death. For most individuals, mild or moderate COVID-19 generally lasts around 2 weeks [2].

Indeed, public health efforts initially focused on reducing the immediate effects of the disease. However, as the pandemic progressed, it became increasingly evident that SARS-CoV-2 infection could lead to persistent physical and mental health problems for several weeks or months, requiring prolonged periods of clinical care and increasing the burden on the healthcare system [3, 4].

This condition aligns with UK clinical guidelines, which differentiate between ongoing symptomatic COVID-19 (manifestations present four to 12 weeks after onset) or post-COVID-19 syndrome (occurring beyond 12 weeks after onset) [5, 6]. To date, there is no consensus on a standard terminology to describe the prolonged symptoms following COVID-19 infection and various terms have been used in the literature. Recent articles have commonly used the terms “long-COVID-19” and “post-acute COVID-19” to describe this condition. According to the World Health Organization (WHO), symptoms that persist for more than 12 weeks after the onset of COVID-19 and in the absence of an alternative diagnosis are considered to be part of the “Long COVID-19” also known as “post-COVID-19 syndrome” (PCS) [7, 3]. This later was first described as a clinical entity in May 2020, when COVID-19 patients were still showing symptoms several weeks after their acute infection and shortly after the first advanced cases appeared. Besides, it is a multi-system disease occurring even in undiagnosed individuals (mild or asymptomatic cases) [8, 9]. The incidence of PCS varies across studies [10–12]. Its exact duration remains unclear and is an area of ongoing research [6, 13].

Research efforts are currently underway to better understand the causes and mechanisms of PCS, as well as to develop effective prevention and treatment strategies [4, 14]. In addition to the organ damage caused by the initial infection, various other potential causes of PCS have been suggested, including occult virus persistence, autoimmune reactions, impaired endothelial function, chronic inflammatory mechanisms, coagulation activation, and damage to the autonomic nervous system [15].

In Tunisia, there are few published studies on PCS, and those that exist are all of a cross-sectional nature [16, 17]. To the best of our knowledge, this is the first cohort study dealing with the PCS in the general Tunisian population.

In this study, we aimed to estimate the incidence of PCS in the Tunisian population and identify its potential risk factors.

## Methods

### Study design

We carried out a cohort study that included mild-to-moderate symptomatic patients diagnosed with COVID-19 from the COVID-19 triage unit of the University Hospital of Monastir between April 2021 and June 2022. The follow-up period was 12 weeks.

### Setting

Monastir is a coastal governorate located in the east of Tunisia, with a population of approximately 600,000 inhabitants. The healthcare system in the governorate is well-established, with several public and private hospitals and clinics. The University Hospital of Monastir is a tertiary care center that serves as a referral hospital for the surrounding regions. Patients have been recruited from the COVID-19 triage unit of the University Hospital of Monastir.

### Participants

Eligible participants were mild-to-moderate symptomatic patients with confirmed diagnosis of COVID-19 by either reverse transcriptase-polymerase chain reaction (RT-PCR) or rapid antigen test during the period from April 1, 2021 to June 30, 2022.

Exclusion criteria were patients who declined to participate, uncooperative patients, patients diagnosed with a mental health problem, patients who were not reachable by phone, or had died.

### Sample size

The required sample size was calculated based on an incidence proportion of PCS of 45% [18] using the following formula: n = (zα/2)^2^ p(1-p)/i^2^ with [z_α/2_ = 1,96, i = 0.05, p = estimated proportion]. The minimum number of subjects required was then 381 patients.

### Data collection

Data collection was made on two times (t_1_ = the initial assessment when attending the COVID-19 triage unit) and (t_2_ = 12 weeks after the date of diagnosis). The initial assessment was through face-to-face interview and the follow-up assessment was performed via telephone.

Data collected at t_1_ were sex, history of chronic diseases (Hypertension, Diabetes, respiratory diseases, cardiovascular diseases, renal diseases, immunosuppression…), other anterior episodes of COVID-19 infection, vaccination status, number of vaccine doses received…

Data collected at t_2_ were duration of the acute COVID-19 illness, secondary hospitalization and post COVID-19 symptoms. The symptoms were categorized into various subgroups including: neurological and psychological symptoms (memory impairment, headache, sleep disorders, depressed mood, Anxiety); Ear, nose, and throat symptoms (Anosmia, Ageusia, vertigo, otalgia); Digestive symptoms (Abdominal pain, diarrhea, nousea/vomiting); rheumatological symptoms syndrome (Arthralgia/ lumbalgia, Myalgia) and dermatological symptoms (hair loss, skin rashes) [16].

### Variable definitions

In our study, PCS was defined by the presence of at least one symptom persisting or present beyond 12 weeks after the onset of acute COVID-19 and not attributable to other diagnoses. This definition has been used in several studies in the literature [3, 5, 18–20].

The diagnosis of PCS was retained if the persistent or the present symptomatology beyond 12 weeks after the onset of acute phase was not reported by the patient before his COVID-19 infection and could not be attributable to other underlying diseases.

Symptoms prior to COVID-19 infection, underlying diseases and comorbidities were searched through patient interview and medical examination.

So far, there is no standardized test to diagnose PCS [21, 22] WHO- Delphi recommendations developed the first consensus of PCS definition [23]. However, PCS diagnosis is very subjective because of the lack of specific symptoms to the condition [21, 22]. Ruling-out other diagnosis includes a complementary lengthy and expensive process of tests that are difficult to achieve in common practice [22].

The viral variant (Alpha, Delta or Omicron) was defined based on the epidemic wave of the virus at the time of diagnosis [24, 25].

### Statistical analysis

The collected data were entered into a Google form and transferred to the SPSS software. Continuous variables were presented as the mean ± standard deviation (SD) or medians and interquartile ranges, depending on the distribution of the variable, and categorical variables as counts and percentages. We calculated the cumulative incidence (Incidence proportion) of post COVID-19 syndrome according to the following formula [26]:

$$Cumulative\ Incidence=\frac{New\ cases\ of\ Post\ COVID\ 19\ syndrome\kern0.5em after\ 12\ week\ follow\ up}{COVID\ 19\ patients\ who\ completed\ the\ 12\ week\ follow\ up}\times 100$$

We compared continuous variables using the Mann-Whitney U test. We compared categorical variables using the chi-square test. The determinants of PCS were identified through multivariate analysis using Poisson regression with robust variance [27, 28]. Covariates selection for the multivariate analysis was based on clinical relevance and *p* value ≤0.20 in univariate analysis. The significance level was set at *p* < 0.05.

### Ethical considerations

The protocol of the study was approved by the ethics committee of the faculty of medicine in Monastir. All participants were informed of the study’s purpose and gave their informed oral consent to participate. Participant anonymity was strictly maintained throughout the data collection and analysis process.

## Results

From April 1, 2021, to June 30, 2022, 4739 patients with mild to moderate symptomatology of COVID-19 infection visited the COVID-19 triage unit of Monastir University Hospital. Among them, 1996 were diagnosed with COVID-19 via reverse transcriptase-polymerase chain reaction (RT-PCR) or rapid antigen test. Of these patients, 1841 gave their consent to participate in the study and responded to the t_1_ assessment. After 12 weeks from the date of diagnosis, 390 of them were unreachable by telephone to answer the t_2_ survey. Thus, our study included 1451 patients diagnosed with COVID-19 infection who responded to the follow-up evaluation for PCS after 3 months.

The respondents had a mean age of 40.7 years (SD = 17.2). Women accounted for 63.3% of the respondents. Chronic conditions were recorded among 406 (28%) of patients. Arterial hypertension and type 2 diabetes were identified in 142 (9.8%) and 123 (8.5%) respectively. Respiratory disease was present in 51 (3.5%) patients, chronic cardiac disease in 39 (2.7%), and renal disease in 17 (1.2%). Among the respondents, 1322 patients (91.1%) reported receiving COVID-19 vaccination, while 119 patients (8.2%) remained unvaccinated. The majority of participants, 1320 (90.9%), did not require hospitalization (Table 1).

**Table 1:** Characteristics of 1451 COVID-19 patients (Monastir, Tunisia; April 2021 - June 2022)

	n	%
**Gender**		
Male	532	36.7
Female	919	63.3
**Age group**		
< 18	96	6.7
18-39	618	42.6
40-64	596	41.0
≥ 65	126	8.7
Not specified	15	1.0
**Chronic conditions**		
Yes	406	70.8
No	1028	28.0
Not specified	17	1.2
**Hypertention**		
Yes	142	9.8
No	1292	89.0
Not specified	17	1.2
**Diabetes**		
Yes	123	8.5
No	1311	90.3
Not specified	17	1.2
**Respiratory disease**		
Yes	51	3.5
No	1383	95.3
Not specified	17	1.2
**Heart disease**		
Yes	39	2.7
No	1395	96.1
Not specified	17	1.2
**Renal disease**		
Yes	17	1.2
No	1417	97.6
Not specified	17	1.2
**Vaccination**		
Vaccinated	1322	91.1
Not vaccinated	119	8.2
Not specified	10	0.7
**COVID-19 infection**		
Before vaccination	750	51.7
After one dose of vaccine	97	6.7
After two doses of vaccine	559	38.5
Not specified	45	3.1
**COVID-19 episodes number**		
One episode only	1266	87.2
Two episodes	172	11.9
Three episodes or more	12	0.8
Not specified	1	0.1
**Hospitalization**		
Yes	114	7.9
No	1320	90.9
Not specified	17	1.2

Among the 1451 patients included for analysis, 639 developed post COVID-19 syndrome. The cumulative incidence of PCS was of 44.03 (95% CI [41.47; 46.58]) per 100 persons among those who completed the follow-up. Individuals diagnosed with PCS revealed a range of persistent symptoms: Fatigue was found to be the most common symptom, present in 312 cases (21.2%). Following behind were cognitive impairment, including memory loss and difficulty concentrating observed in 150 patients (10.3%), then dyspnea reported by 117 patients (8.1%) and headache by 97 patients (6.7%). Cough, arthralgia, anosmia, and chest pain were respectively reported by 84 patients (5.8%), 61 patients (4.2%), 55 patients (3.8%) and 49 patients (3.4%) (Table 2).

**Table 2:** Clinical manifestations of post-COVID syndrome after 12-Week Follow-Up among 1451 patients diagnosed between April 2021 and June 2022 in Monastir, Tunisia

	n	Percentage
**Fatigue**	312	21.5
**Cardiovascular and respiratory symptoms**		
Dyspnea	117	8.1
Cough and/or expectoration	84	5.8
Chest pain	49	3.4
Thrombophlebitis	3	0.2
**Ear, nose, and throat symptoms**		
Anosmia	55	3.8
Ageusia	31	2.1
Vertigo	9	0.6
Otalgia	6	0.4
**Neurological and psychological symptoms**		
Memory impairment	150	10.3
Headache	97	6.7
Sleep disorders	20	1.4
Depressed mood	17	1.2
Anxiety	13	0.9
**Digestive symptoms**		
Abdominal pain	10	0.7
Nausea/vomiting	2	0.1
Diarrhea	2	0.1
**Rheumatological symptoms**		
Arthralgia/lumbalgia	61	4.2
Myalgia	8	0.5
**Dermatological symptoms**		
Hair loss	15	1.0
Skin rashes	2	0.1

**Fig. 1:**
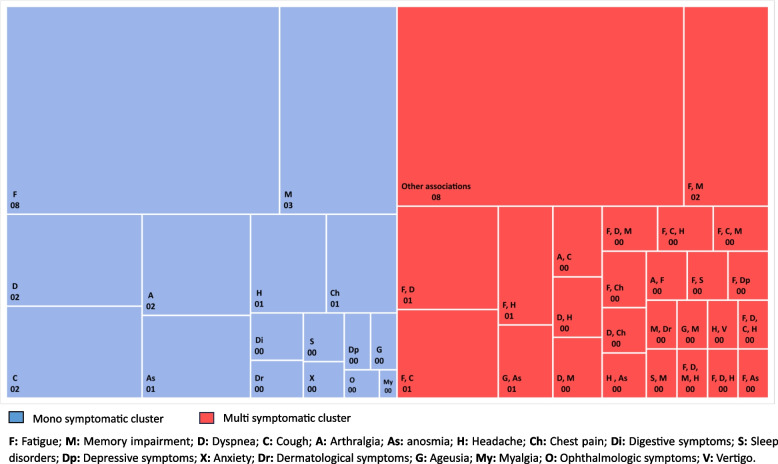
Percentages of the main Post COVID-19 symptoms after 12-Week Follow-Up among 1451 patients diagnosed between April 2021 and June 2022 in Monastir, Tunisia

One out of two peoples with post COVID-19 syndrome had two or more persistent symptoms after 3 months of follow-up. The main recorded associations of symptoms were fatigue and memory impairment (2.3%), fatigue and dyspnea (1.4%) and fatigue and cough (1.2%) (Fig. 1).

**Fig. 2:**
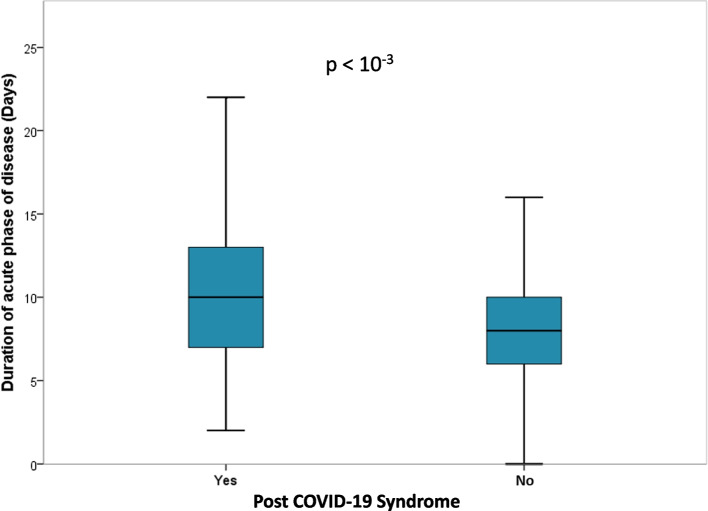
Comparison of duration of the acute phase of disease between groups with and without post COVID-19 Syndrome among 1451 patients diagnosed between April 2021 and June 2022 in Monastir, Tunisia

Individuals who developed PCS had a significantly longer duration of acute illness compared to those who did not. Specifically, the median duration was 10 days (range: 7 to 13 days) in the PCS group, whereas it was 8 days (range: 6 to 10 days) in the non-PCS group (*p* < 10^−3^) (Fig. 2).

The univariate analysis showed a statistically significant association between the development of PCS and various factors, including age, female gender, presence of comorbidities, lack of vaccination, longer duration of acute illness, hospitalization, history of previous infections with COVID-19, and the viral variant, as presented in Table 3.

**Table 3:** Factors associated with post-COVID-19 syndrome among 1451 patients diagnosed between April 2021 and June 2022 in Monastir, Tunisia (Univariate analysis)

	N	PCS (n (%))	*p*	c RR	CI (95%)
**Age**					
< 18	96	32 (33.3)	< 10^−3^	1	
18-39	618	236 (38.2)		1.14	[0.85 - 1.54]
40-64	596	310 (52.0)		1.56	[1.16 – 2.09]
≥ 65	126	52 (41.2)		1.25	[0.88 - 1.77]
**Gender**					
Male	532	185 (34.8)	< 10^-3^	1	
Female	919	454 (49.4)		1.42	[1.25 - 1.62]
**Comorbidities**					
No	1028	402 (39.1)	< 10^−3^	1	
Yes	406	228 (56.2)		1.43	[1.28 - 1.61]
**Hypertention**					
No	1292	555 (43.0)	0.025	1	
Yes	142	75 (52.8)		1.23	[1.04 - 1.45]
**Diabetes**					
No	1311	570 (43.5)	0.25	1	
Yes	123	60 (48.8)		1.12	[0.92 - 1.35]
**Respiratory disease**					
No	1383	598 (43.2)	0.006	1	
Yes	51	32 (62.7)		1.45	[1.16 – 1.80]
**Heart disease**					
No	1395	612 (43.9)	0.77	1	
Yes	39	18 (46.2)		1.05	[0.74 - 1.48]
**Renal disease**					
No	1417	622 (43.9)	0.79	1	
Yes	17	8 (47.1)		1.07	[0.64 - 1.78]
**Two or more doses of vaccine before COVID-19 infection**					
No	847	427 (50.4)	< 10^−3^	1	
Yes	559	198 (35.4)		0.50	[0.47 – 0.53]
**Hospitalization**					
No	1320	560 (42.4)	< 10^−3^	1	
Yes	114	73 (64.0)		1.51	[1.29 - 1.75]
**Number of COVID-19-episodes**					
Only one	1266	521 (41.2)	< 10^-3^	1	
Two episodes	172	109 (63.4)		1.54	[1.35 - 1.75]
Three episodes or more	12	9 (75.0)		1.82	[1.30 - 2.54]
**Viral variant**					
Omicron	651	231 (35.5)	< 10^−3^	1	
Delta	459	235 (51.2)		1.44	[1.25 - 1.65]
Alpha	326	162 (49.7)		1.40	[1.20 - 1.60]

*PCS* Post Covid Syndrome, *cRR* crude Relative Risk, *CI (95%)* Confidence Interval (95%)

Multivariate analysis revealed that the main predictors of PCS were female gender (RR = 1.54; CI95% [1.30 - 1.82]), pre-existing comorbidities (RR = 1.30; CI95% [1.10 - 1.52]), duration of acute COVID-19 illness (days) (RR = 1.02; CI95% [1.01 - 1.03]), hospitalization (RR = 1.27; CI95% [1.05 - 1.53]), number of COVID-19 episodes (RR = 1.46; CI95% [1.28 – 1.67]) and patients having receive two or more doses of vaccine before COVID-19 infection (RR = 0.82; CI95% [0.70 - 0.96]) (Table 4).

**Table 4:** Risk Factors of Post COVID-19 Syndrome among 1451 patients diagnosed between April 2021 and June 2022 in Monastir, Tunisia (Multivariate analysis)

	*p*	aRR	CI (95%)
Female gender	< 10^−3^	1.54	[1.30 - 1.82]
Comorbidities^a^	0.002	1.30	[1.10 - 1.52]
Duration of acute phase of disease (days)	< 10^−3^	1.02	[1.01 - 1.03]
Hospitalization	0.012	1.27	[1.05 - 1.53]
Number of Covid-19 episodes	< 10^−3^	1.46	[1.28 – 1.67]
Two or more doses of vaccine before Covid-19 infection	0.016	0.82	[0.70 - 0.96]

*aRR* adjusted Relative Risk, *CI (95%)* Confidence Interval (95%)

^a^Comorbidities: The presence of at least one of these chronic conditions: Hypertension, Diabetes, respiratory diseases, cardiovascular diseases, renal diseases, immunosuppression…)

## Discussion

Our study contributes to the growing body of evidence on PCS, estimating its incidence in the Tunisian population, and highlighting its symptoms, and its predictive factors. To the best of our knowledge, the limited number of published national studies is essentially cross-sectional in nature [16, 17]. Our study is therefore the first Tunisian cohort study of PCS in the general Tunisian population. The overall incidence was found to be high at 44%, with the most frequently reported symptoms were fatigue, with an incidence of 21.5%, followed by cognitive impairment (10.3%), including memory loss and difficulty concentrating, dyspnea (8%), headache (6.6%), cough (5.8%), arthralgia (4.2%) and anosmia (3.8%). Our findings indicate that PCS is significantly predicted by female gender, comorbidities, hospitalization, duration of acute COVID-19 illness, and number of COVID-19 episodes.

Our cohort consisted of mild to moderate COVID-19 cases, where the majority (90.9%) not requiring hospitalization. After 3 months from the initial symptom onset, PCS was observed in 44% (95% CI [41.47 - 46.58]) of these patients.

The majority of research has centered on PCS among COVID-19 patients who were hospitalized, examining the condition at 1 to 2 or 6 months after the onset of symptoms [11, 13, 29–31].

Cohort studies involving COVID-19 patients who were previously admitted during the acute phase yielded the following incidences of PCS: 76% (1876/2469) 6 months after admission in China [30], 49.6% (267/538) 3 months after discharge in the same country [31], 74% (81/110) within 2-3 months after admission in the UK [11], and 87% (124/143) 2 months after discharge in Italy [13].

In contrast, studies focused on mild to moderate COVID-19 outpatients reported the subsequent frequencies of PCS: 53.0% (304/573) after 12 months of symptom onset in Italy [32], 53.1% (108/203) after 4 months (125 days) in the Faroe Islands [33], 46.3% (99/214) during a 6-week follow-up [34], 14.2% (55,663 / 391,990) after 3 months, 27.8% (123/442) after 4 months, and 34.8% (123/353) after 7 months in Germany [29, 35], and 45% (227/504) after 3 months after diagnosis in Saudi Arabia [18].

Upon examining the available national literature, a cross-sectional study was carried out in Tunisia during February 2022. The study involved the general population through an online platform and a self-administered questionnaire. The results of this investigation revealed a PCS prevalence of 46.5%, which aligns with the incidence observed in our research. Nevertheless, it is imperative to recognize that a direct comparison between incidence and prevalence may not be entirely appropriate due to differences in study design [16].

Additionally, a systematic review and meta-analysis revealed that the global prevalence of PCS was estimated at 0.37 0.37 (95% CI, 0.26-0.49), 0.25 (95% CI, 0.15-0.38), 0.32 (95% CI, 0.14-0.57), and 0.49 (95% CI, 0.40-0.59) at 30-, 60-, 90-, and 120-days post-infection, respectively [36]. Besides, this systematic review provided separate estimates for inpatients and outpatients, with prevalence rates of 0.54 (95% CI, 0.44-0.63) and 0.34 (95% CI, 0.25-0.46), respectively [36].

Therefore, it is quite clear that the incidence of PCS may vary depending on several factors, including the definition and criteria used to diagnose the syndrome, the time elapsed since COVID-19 infection (ranging from 1 to 12 months), the severity of the infection, the need for hospitalization and the characteristics of the study population [32, 36].

In agreement with other evidence, our study identified and characterized the most commonly reported symptoms among individuals affected by PCS, which included fatigue (21.5%), cognitive impairment (10.3%), dyspnea (8%), headache (6.7%) and cough (5.8%), keeping in line with pertinent research both at the national and global levels [16, 37–39]. Indeed, a recent meta-analysis reported that the most commonly reported symptoms were fatigue, dyspnea, sleep disorder, and difficulty concentrating (32, 25, 24, and 22%, respectively, at 3- to < 6-month follow-up) [37]. Besides, findings from a further recent meta-analysis indicate that a substantial proportion of individuals continue to experience persistent fatigue beyond 12 weeks following COVID-19 diagnosis (0.32, 95% CI: 0.27-0.37), and exhibit cognitive impairment as well (0.22, 95% CI: 0.17-0.28) [38].

Moreover, two updated systematic review on PCS and its lasting impact have shed light on the prevalent clinical manifestations that persist after the acute phase of the disease. The review underscores that fatigue (54.11%) and insomnia (25.98%) were the most frequently reported persistent symptoms among patients, reflecting the considerable challenge the disease poses to the physical and mental well-being of those affected [40, 41].

It’s important to highlight that as of the time of writing this article, the exact pathophysiology of PCS remains incompletely understood. However, immune dysregulation, persistent inflammatory responses, autoimmune response, reactivation of pathogens, and changes in the host’s microbiome are believed to potentially play a potential role in the onset of PCS [9].

Thanks to a growing body of literature, the manifestations of PCS are increasingly obvious. Hence, it becomes crucial to raise awareness of the symptoms of PCS among healthcare professionals. The objective of these efforts is to detect early PCS indicators and offer appropriate therapeutic interventions. Additionally, we propose the establishment of comprehensive rehabilitation services aimed at effectively managing these symptoms and optimizing the functional recovery of COVID patients. These services should be provided by a proficient multidisciplinary team over an extended period, tailored to address the specific needs of these individuals [42]. Lastly, it is strongly recommended to create a structured and validated PCS questionnaire to thoroughly delineate the complete clinical spectrum and enhance the reliability and reproducibility of studies [32]. Nevertheless, more research is needed to fully understand the long-term effects of the virus and the range of symptoms that may occur.

Furthermore, our study identified several risk factors associated with PCS, including female gender, comorbidities, hospitalization, duration of the acute phase of COVID-19, number of COVID-19 episodes and number of vaccine doses received before infection. Similarly, two meta-analyses have identified that female sex is an independent prognostic factor [43, 44]. Indeed, many studies suggested that female hormones as well as high IgG antibodies production among women may prolong the hyper inflammatory status resulting in PCS [45, 46]. Besides, our study confirmed that hospitalized patients are more likely to develop PCS, keeping in line with previous research [47, 48]. This association could be attributed, on one hand, to the severity of hospitalized cases, which is linked to significant immune responses and excessive cytokine reactions, causing organ damage [49]. On the other hand, it could be due to extended periods of immobility or the use of mechanical ventilation, but this susceptibility is not exclusive to COVID-19. Consequently, individuals in this category are likely to experience extended recovery periods [29].

Interestingly, based on our findings, the duration of the acute phase of the disease emerged as a predictive factor of developing PCS. Notably, people with a prolonged acute phase were at higher risk; each increase of 1 day in the duration of the illness multiplies the risk of developing PCS by 1.02 with a confidence interval of [1.01-1.03]. To our knowledge, no previous study has explored the relationship between the duration of the acute phase of the disease and the development of PCS. However, two meta-analyses have shown that the severity of the acute phase and the fact of having several symptoms at the same time constitute a risk factor for the development of this syndrome [43, 44].

In addition, a higher risk of PCS was reported among individuals with preexisting comorbidities. Consistently, a recent meta-analysis published in 2023 revealed that preexisting comorbidities were significantly associated risk factors for developing PCS. These comorbidities included anxiety and/or depression (OR, 1.19; 95% CI, 1.02 to 1.40; I^2^ = 96%), asthma (OR, 1.24; 95% CI, 1.15 to 1.35; I^2^ = 53%), chronic kidney disease (OR, 1.12; 95% CI, 0.98 to 1.28; I^2^ = 22%), chronic obstructive pulmonary disease (OR, 1.38; 95% CI, 1.08 to 1.78; I^2^ = 77%), diabetes (OR, 1.06; 95% CI, 1.03 to 1.09; I^2^ = 0%), immunosuppression (OR, 1.50; 95% CI, 1.05 to 2.15; I^2^ = 0%), and ischemic heart disease (OR, 1.28; 95% CI, 1.19 to 1.38; I^2^ = 0%) [50].

These predictors suggest a complex interplay of biological, environmental, and clinical factors in the development of PCS. The identification of these factors offers crucial insights into the underlying mechanisms of PCS and may help inform targeted interventions for prevention and management of this debilitating condition. Moreover, understanding these risk factors can help clinicians and public health officials identify individuals who may be at higher risk of developing post-COVID syndrome and provide targeted support and management strategies. Indeed, risk factors knowledge are needed to guide management programs and infrastructure and will enable maximizing available resources among high-risk population. It might also allow doctors to be better prepared to take care of these patients [50]. In addition, risk factors knowledge will help raising awareness among targeted COVID-19 patients of persistent physical and psychosocial consequences possibility [44].

In the light of our study results, we recommend systematic specialist follow-up consultations between two and 3 months after the acute phase of COVID-19 for people with the risk factors mentioned. The aim of these measures is to identify early signs of PCS and provide appropriate therapeutic interventions.

Contrariwise, two or more COVID-19 vaccine doses was found to be a protective factor from long COVID-19 symptoms. Consistently, A UK cohort study of 28,356 participants who received at least one dose of adenovirus vector or COVID-19 mRNA vaccine after testing positive for SARS-CoV-2 infection showed a decreased probability (− 12.8%) of lingering symptoms after first dose COVID-19 vaccination and a sustained improvement (− 8.8%) after a second dose [51]. Further previous published studies suggested that immunization reduce inflammatory responses leading to lower rates of PCS [52, 53]. Hence, it is important to promote vaccination against COVID-19 and to emphasize the benefits of receiving two or more doses of vaccine for the general population, and in particular for individuals at risk.

Our study presents several notable strengths. Firstly, the large sample size and a substantial number of relevant outcomes provide statistical power to our analysis. Secondly, we employed a prospective data collection method which effectively mitigates the potential for recall bias, a common concern in retrospective studies. Thirdly, we used validated diagnostic tests, including Rapid Diagnostic Test and Polymerase Chain Reaction (PCR) to confirm COVID-19 diagnoses, thus enhancing the precision and reliability of our results. These strengths collectively contribute to the robustness and validity of our study’s findings.

Despite the valuable insights provided by our study, some limitations should be considered. First, our study was conducted in a single center, which may limit the generalizability of our findings to other regions or populations. Second, the study relied on self-reported which may be subject to measurement bias. Third, our follow-up period was limited to 3 months, which may not capture the long-term effects of PCS. Fourth, the study did not explore the involvement of other factors in the onset of the syndrome, such as socio-economic factors, smoking, obesity and symptoms initially developed during the acute phase of the infection [32].

Considering the high incidence of PCS, there is a need for further research concerning this condition and its potential long-term consequences. Future studies should aim to identify mechanisms of PCS and develop effective treatments and management strategies to improve the quality of life of affected individuals.

## Conclusion

This study adds to the growing body of evidence on PCS, demonstrating a high incidence (44%) among the Tunisian population. These results are consistent with other studies worldwide, highlighting the urgent need for increased attention and research into this emerging health issue. The identification of risk factors associated with PCS, such as female gender, comorbidities, hospitalization, duration of the acute phase of COVID-19, number of COVID-19 episodes and number of vaccine doses received before infection could help healthcare professionals in developing effective preventive and management strategies. Moreover, further research is crucial to understanding the underlying mechanisms of this condition and to developing targeted interventions aimed at improving outcomes for those affected by PCS.

## Data Availability

Data are available from the corresponding author on reasonable request.
